# Single‐Cell Transcriptomic Analysis of Primary and Metastatic Tumor Ecosystems in Esophageal Squamous Cell Carcinoma

**DOI:** 10.1002/advs.202204565

**Published:** 2023-01-29

**Authors:** Yongxu Jia, Baifeng Zhang, Chunyang Zhang, Dora Lai‐Wan Kwong, Zhiwei Chang, Shanshan Li, Zehua Wang, Huiqiong Han, Jing Li, Yali Zhong, Xin Sui, Li Fu, Xinyuan Guan, Yanru Qin

**Affiliations:** ^1^ Department of Clinical Oncology The First Affiliated Hospital Zhengzhou University Zhengzhou 450052 P. R. China; ^2^ Departments of Clinical Oncology The University of Hong Kong‐Shenzhen Hospital Shenzhen 518009 P. R. China; ^3^ Departments of Clinical Oncology Li Ka Shing Faculty of Medicine The University of Hong Kong Hong Kong P. R. China; ^4^ Department of Thoracic Surgery The First Affiliated Hospital Zhengzhou University Zhengzhou 450052 P. R. China; ^5^ Guangdong Provincial Key Laboratory of Regional Immunity and Diseases Department of Pharmacology and International Cancer Center Shenzhen University Health Science Center Shenzhen 518060 P. R. China; ^6^ Advanced Energy Science and Technology Guangdong Laboratory Huizhou 528200 China

**Keywords:** bioinformatics, esophageal squamous carcinoma, immunofluorescence and immunohistochemistry, lymph node metastatic microenvironment, single cell RNA sequencing

## Abstract

Lymph node metastasis, the leading cause of mortality in esophageal squamous carcinoma (ESCC) with a highly complex tumor microenvironment, remains underexplored. Here, the transcriptomes of 85 263 single cells are analyzed from four ESCC patients with lymph node metastases. Strikingly, it is observed that the metastatic microenvironment undergoes the emergence or expansion of interferon induced *IFIT3* ^+^ T, B cells, and immunosuppressive cells such as *APOC1* ^+^ *APOE* ^+^ macrophages and myofibroblasts with highly expression of immunoglobulin genes (*IGKC*) and extracellular matrix component and matrix metallopeptidase genes. A poor‐prognostic epithelial‐immune dual expression program regulating immune effector processes, whose activity is significantly enhanced in metastatic malignant epithelial cells and enriched in *CD74* ^+^ *CXCR4* ^+^ and major histocompatibility complex (MHC) class II genes upregulated malignant epithelia cells is discovered. Comparing with primary tumor, differential intercellular communications of metastatic ESCC microenvironment are revealed and furtherly validated via multiplexed immunofluorescence and immunohistochemistry staining, which mainly rely on the crosstalk of *APOC1* ^+^ *APOE* ^+^ macrophages with tumor and stromal cell. The data highlight potential molecular mechanisms that shape the lymph‐node metastatic microenvironment and may inform drug discovery and the development of new strategies to target these prometastatic nontumor components for inhibiting tumor growth and overcoming metastasis to improve clinical outcomes.

## Graphical abstract

Here not only a high‐resolution landscape of the tumor, immune, and stromal compartments is provided in metastatic lymph node but also metastatic‐specific patterns comparing with primary tumor are highlighted, which provide insights that may help to shape the tumor microenvironment and inform the development of new strategies against these prometastatic nontumor components for inhibiting tumor growth and overcoming metastasis.

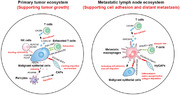

## 1 Introduction

Metastasis is a nonrandom, coordinated progression of cancer and has been implicated in the most common cause for cancer mortality. Lymph node (LN) metastasis is one of major determinants of clinical staging and prognosis in malignant tumors, especially esophageal squamous cell carcinoma (ESCC). The tropism for metastasis is due to the bilateral interaction between the primary tumor (PT) and distinct organs.^[^ ^1^ ^]^ For instance, the chemokine receptors CXCR4 expressed in breast cancer binds to the chemokine ligands CXCL12 in lymph nodes metastasis, but binds to CCL21 in lung metastasis.^[^ ^2^ ^]^ Furthermore, premetastatic niche has been established and educated in distant organ prior to the arrival of primary tumor cells to support future metastatic outgrowth by recruiting bone marrow‐derived cells and releasing cytokines.^[^ ^1^ ^]^ The lymph nodes serve as the main repositories for immune cells residing such as lymphocytes, myeloid cells, and fibroblasts. Compared with primary tumors, metastatic lymph node immune microenvironment is more complex with unexplored mechanisms. Functional exhausted T cells, density decreased dendritic cells, domesticated myeloid cells, and fibroblasts have been reported to play important roles in premetastatic niche formation.^[^ ^3^, ^4^ ^]^ However, the mechanism underlying the formation of the lymph node metastatic microenvironment and organ‐specific metastatic tropism in ESCC remain underexplored.

Single‐cell RNA sequencing (scRNA‐seq) offers an extraordinary opportunity to address these questions by dissecting the intratumor heterogeneity of ESCC and its tumor microenvironment (TME). In recent years, several studies have successfully obtained insight into the collective behavior of tumor cell communities in ESCC by applying this technique to human ESCC primary tumor tissues and their adjacent normal tissues.^[^ ^5^, ^6^, ^7^, ^8^, ^9^ ^]^ Here, we profile the transcriptome of 85 263 single cells from four ESCC patients with lymph node metastases and generate an atlas of the whole metastatic TME. In addition, we also investigated the prognostic value of these metastatic related cell subsets or expression signatures by correlating our scRNA‐seq data with published bulk RNA sequencing profiles. These results advance the understanding of establishing a metastatic TME and provide a basis for personalized treatment of ESCC patients with metastasis.

## 2 Experimental Section

### 2.1 Patient Samples

**Figure 1:**
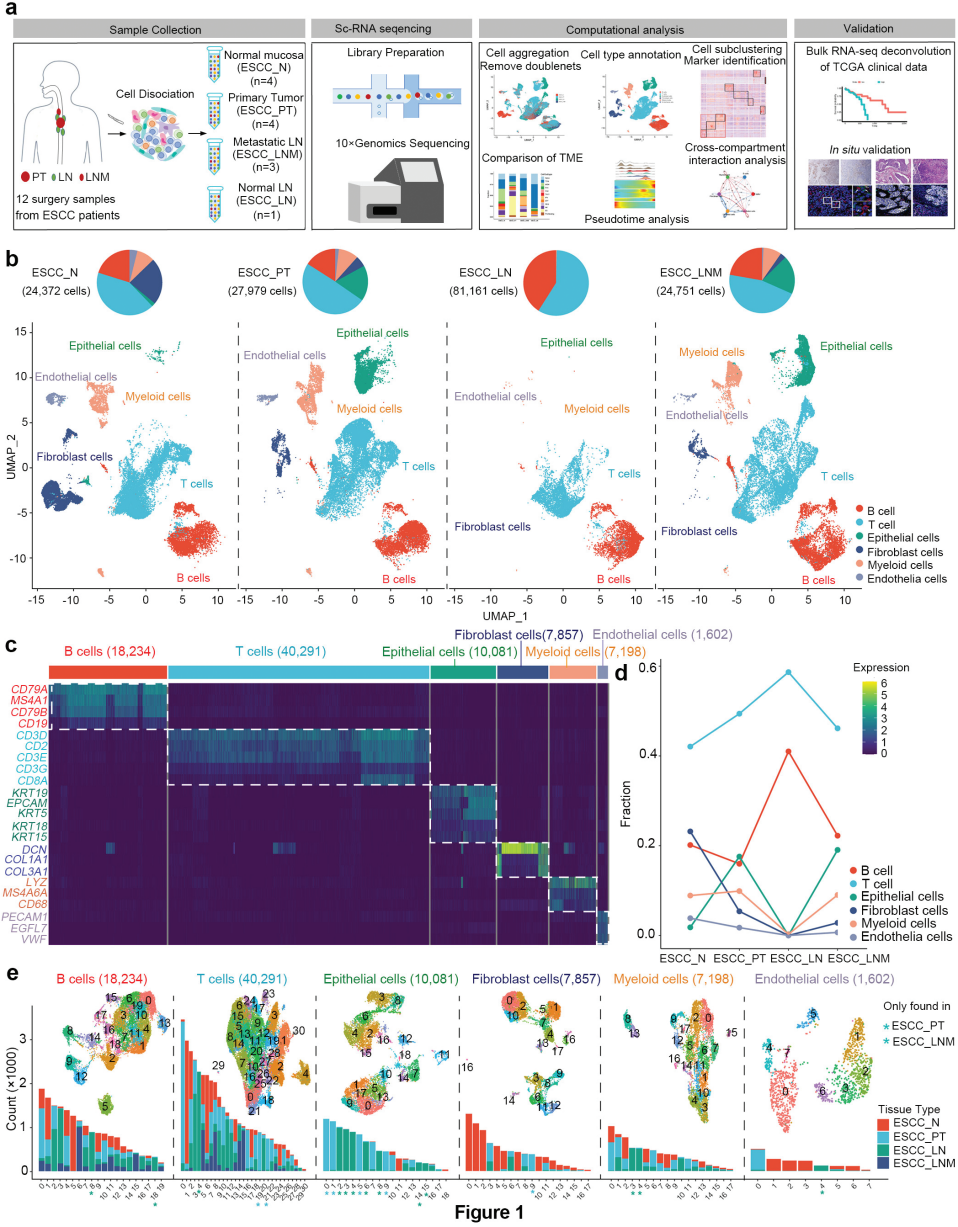
The single‐cell transcriptional landscape of ESCC in normal adjacent, primary tumor, metastatic, and normal lymph node ecosystem. a) Overview of the study design and workflow. b) Uniform manifold approximation and projection (UMAP) of 85 263 cells showing the components of the normal adjacent, primary tumor, metastatic, and normal lymph node microenvironment, color‐coded by the major cell lineage. c) A heatmap shows cell lineage‐specific marker genes (rows) that are differentially expressed across ESCC cells (columns). Yellow: high expression and dark purple: low expression. d) The dynamic changes of fraction of major cell lineages across four distinct ESCC tumor microenvironment. e) UMAP plot and cells number of subclusters for each major cell lineage, color‐coded by their associated subclusters and originated sources.

Four patients with pathologically diagnosed of ESCC were enrolled in this study, and none of patient received preoperative treatment. 12 independent surgical resected specimens were collected from four ESCC patients, including PT tissue, adjacent nontumor tissue (N) and lymph node with metastasis (LNM, three cases) or without metastasis (LN, one case), and then were all fresh‐processed for scRNA‐seq (**Figure** **1**a). All clinical samples were collected at the First Affiliated Hospital of Zhengzhou University (Zhengzhou, China) and their clinical information are summarized in Table S1 in the Supporting Information. Informed consent was obtained from all patients before the collection of samples and samples used in this study were approved by the Committees for Ethical Review of Research at Zhengzhou University (2022‐KY‐0149).

### 2.2 Single‐Cell Suspensions, Library Construction, and Sequencing

The fresh tissue samples surgical resected from ESCC patients were rinsed with phosphate buffered saline (PBS) on ice and then was placed into the Gel Bead and Multiplex Kit and Chip Kit (10× Genomics, Pleasanton, CA, USA) was used to construct a chromium single‐cell 3′‐library according to the manufacturer's instructions. Libraries were sequenced on an Illumina HiSeq X Ten system, and mapped to the human reference genome (build GRCh38) using CellRanger‐4.0.0 (10× Genomics).^[^ ^10^ ^]^

### 2.3 Single‐Cell RNA‐Seq Data Processing

Beginning with raw base call (BCL) files generated by Illumina HiSeq X Ten system, CellRanger‐4.0.0 package with “cellranger mkfastq” commend was used to demultiplex the BCLs files to generate raw FASTQs. By using “cellranger count” commend, raw FASTQs files was aligned to the human reference genome (build GRCh38), filtered, barcode counted and unique molecular identifier (UMI) counted to generate single cell gene expression profile. To remove the bias, the “cellranger aggr” commend was used to aggregate the gene expression count of all samples from “cellranger count” to normalize those counts to the same sequencing depth and then to recompute the feature‐barcode single cell gene expression matrices.

The output from “cellranger aggr” was converted to a Seurat object using the R Seurat package (version 4.0.1)^[^ ^11^ ^]^ for downstream analysis. Single‐Cell Remover of Doubelts (Scrublet)^[^ ^12^ ^]^ was applied with default parameters to infer and remove cell doublets in each sample individually. To remove low quality cells, the various metrics distribution of the data was visualized and the following thresholds were applied: nUMI > = 300, nGene > = 200, log10GenesPerUMI > 0.8 and mitoRatio < 0.2. To avoid the reduction of average expression for a cell caused by genes with zero count or only expressed in a handful of cells, genes are kept only which are expressed in ten or more cells. The remaining expression matrix was normalized to account for differences in sequencing depth per cell for each sample with “NormalizeData” function in Seurat package. By regressing out cell‐cycle phage (S, G2/M) scores, mitochondrial expression with CellCycleScoring and SCTransform function in Seurat package, the normalized matrix was further scaled to eliminate cell‐cycle and mitochondrial effects and to generate a final single cell gene expression matrix with high quality.

### 2.4 Determination of Cell Subtypes and Identification of Their Marker Gene Signature

RunPCA and RunUMAP function implemented in Seurat were also used to reduce dimensionality and remove batch effect. Cell clusters were initially identified by a default resolution of 0.8 using “FindClusters” function implemented in Seurat. The clusters were annotated as six major cell types based on their average gene expression of canonical markers, including B cells (*CD79A*, *MS41*, *CD79B*, and *CD19*), T cells (*CD3D*, *CD2*, *CD3E*, *CD3G* and *CD8A*), epithelial cells (*KRT5*, *KRT15*, *KRT18*, *KRT19*, and *EPCAM*), fibroblast cells (*DCN*, *COL1A1*, and *COL3A1*), myeloid cells (*LYZ*, *MS4A6A*, and *CD68*), and endothelial cells (*PECAM1*, *EGFL7*, and *VWF*).

To further identify sub‐clusters and annotate them as cell subtypes in each major cell type, the abovementioned step (normalization, dimensionality reduction, and clustering) was repeated for each major cell type. To define marker genes for each sub‐cluster within six major cell types, the FindAllMarkers function implemented in Seurat with default two‐sided nonparametric Wilcoxon rank sum test was used to compare all genes expression files of one sub‐cluster with those of the other sub‐clusters, which will generate significant differentially expressed genes with Bonferroin‐adjusted *P*‐value lower 0.05 and an average natural logarithm fold‐changes of expression at least 0.25 and expression in above 10% of corresponding subcluster. Genes functionally related with cell type or state via literature review was used to annotate the cell subtype of subclusters in each major cell type. The top50 genes ranked by their average fold‐changes of expression for the corresponding cell subcluster was selected as the marker gene signature of this corresponding cell subcluster (Data S2 and S3, Supporting Information).

### 2.5 Copy Number Variants (CNV) Estimation

To explore the heterogeneity of copy number alterations existing in epithelial cells of primary tumor and lymph node metastasis site of ESCC, inferCNV (https://github.com/broadinstitute/inferCNV) was applied to estimate the changes of somatic alterations of large‐scale chromosomal copy number variants (amplifications or deletions) in a single epithelial cell. The epithelial cell raw single‐cell gene expression data were extracted from the Seurat object followed by the software manual. Single cell data from epithelial cells in normal tissue were used as a control reference and then the inferCNV analysis with the default parameters was performed.

### 2.6 Gene Expression Programs (GEPs) of Intratumoral Heterogeneity

To understand the organization of epithelial cells from different locations, based on genes*cells expression matrix from the mixed single‐cell profiles, consensus non‐negative matrix factorization (cNMF) was used to deconvolute different GEPs associated with both cell identity, states, and cellular activities including the hypoxia and cell‐cycle, in additional to their relative usages in each cell.^[^ ^13^ ^]^ To determine the optimal number of GEPs components (*K* = 9), the trade‐off between the stability of the solution and the forbenius reconstruction error for different *K* values (from 2 to 19) was considered. The top50 genes ranked by their contribution scores to specific GEP was selected as the representative gene set to perform pathway enrichment analysis and calculation of GEP score in the cancer genome atlas (TCGA)‐ESCC (*n* = 96) cohort (Data S4 and S5, Supporting Information).

### 2.7 Pathway Enrichment Analysis

To understand the potential function and mechanism of selected gene list such as the marker signature genes of clusters, the enrichment of selected gene list with Gene Ontology terms (C5:BP MSigDB^[^ ^14^ ^]^) using *goseq* R packge^[^ ^15^ ^]^ (false discovery rate (FDR)‐adjusted *P* < 0.05 was considered significant) was assessed. To explore the difference of signaling pathway enrichment between two subclusters, the fgsea package^[^ ^16^ ^]^ with default parameters in a preranked gene list with fold changes was applied to test the enrichment of the 50 hallmark pathways (H: MSigDB) and implemented in the ClusterProfiler package.^[^ ^17^ ^]^

### 2.8 Defining the Signature Score of Cell Subpopulations and GEPs in TCGA‐ESCC Cohort and Their Prognostic Analysis

Based on public batch effects normalized mRNA expression profile from TCGA‐ESCC bulk RNA sequencing data (Esophagus Squamous Cell Carcinoma, 96 patients) (https://api.gdc.cancer.gov/data/3586c0da‐64d0–4b74‐a449–5ff4d9136611), the marker gene signature of different subtypes in six major cell types and GEPs of Epithelial cells was used to estimate their activity in TCGA‐ESCC cohort patients by using Gene set variation analysis (GSVA) in the GSVA package^[^ ^18^ ^]^ (version 1.38.2) (Data S2–S5, Supporting Information). The mean value of activity was used to classify the TCGA‐ESCC patients into high expression and low expression two subgroups and then matched overall survival (OS) of patients from the TCGA Pan‐cancer Clinical Data Resource^[^ ^19^ ^]^ was used to analyze two subgroup patients’ clinical outcome. Kaplan–Meier curves was plotted by using R function *ggsurvplot* and Chi‐squre test statistics were computed using log‐rank tests implemented in the *survival* package.

### 2.9 Developmental Trajectory Analysis

To determine the process of potential functional changes and lineage differentiation of immune cells, the monocle (version 2.18.0) algorithm^[^ ^20^ ^]^ was applied in inferring the potential developmental trajectory of T cells, B cells, Myeloid‐derived Macrophage, and dendritic cells. The expression data of indicated cell type outputted from Seurat were converted to CellDataSet object as the input of Monocle2, and then DDRTree‐based dimension reduction and cells ordering was performed with the significant differentially expressed genes between subclusters (calculated in FindAllMarkers function of Seurat). Finally, differentiation trajectory and pseudotime of cells was inferred and plotted with the default parameters of Monocle2.

### 2.10 Calculation of Functional Module Scores and Correlation Analysis

The scores of functional modules including proliferation, exhaustion, inhibitory, cytotoxicity, antigen processing and presentation, IL2R, costimulatory, apoptosis and immune suppressive score were calculated for each cell of subcluster of interest by using the AddModuleScore function in Seurat. The involved gene features of each module were downloaded from previous study^[^ ^21^ ^]^ and their average expression of single cells for each subcluster were calculated and subtracted by the aggregated expression of randomly selected control gene feature sets. To explore the association between functional module score and pseudotime of cells, their pearson correlation value and *P*‐value were also calculated.

### 2.11 Crosstalk Analysis of Tumor Ecosystems

To understand the crosstalk among cells in the tumor ecosystems, CellChat^[^ ^22^ ^]^ tool was applied to quantitatively infer and analyze the potential interactions across different cell types in the ESCC tumor microenvironment, including interactions among ligands, receptors, and their cofactors. By using CellChat with recommended parameters, cellular communication probability was quantized and the statistically and biologically significant cellular communications were inferred. The strength number of significant cellular communications among tumor ecosystems and dynamic changes between normal tissue, primary tumor, lymph node with metastasis or not were visualized and compared. Detailed significant differentially communicated ligand–receptor pairs among tumor ecosystems in primary tumor versus normal and lymph node metastasis versus primary tumor were also tested based on communication probability and *P*‐value from one‐sided permutation test.

### 2.12 Multiplexed Immunofluorescence (IF) Staining

Multiplex immunofluorescence staining was conducted using the Akoya OPAL Polaris 7‐Color Automation immunohistochemistry (IHC) kit (NEL871001KT). Formalin‐fixed paraffin‐embedded (FFPE) tissue slides were first deparaffinized in a BOND RX system (Leica Biosystems) and then incubated sequentially with primary antibodies targeting CD163 (Abcam, ab182422, 1:500), CD68 (Abcam, ab213363, 1:1000), PD‐1 (CST, D4W2J, 86163S, 1:200), PD‐L1 (CST, E1L3N, 13684S, 1:400), CD3 (Dako, A0452, 1:1), CD4 (Abcam, ab133616, 1:100), CD8 (Abcam, ab178089, 1:200), CD56 (Abcam, ab75813, 1:1000), CD20 (Dako, L26, IR604, 1:1), FOXP3 (Abcam, ab20034, 1:100), The apolipoprotein E (*APOE*) (Abcam, ab183597, 1:100), APOC1 (Abcam, ab198288, 1:100), IFIT1 (proteintech, 23247‐1‐AP, 1:100), IFIT3 (proteintech, 15201‐1‐AP, 1:100), *α*‐smooth muscle actin (SMA) (proteintech, 67735‐1‐Ig, 1:100), CD90 (proteintech, ab133350, 1:100), NDUFA4L2 (proteintech, 16480‐1‐AP, 1:100), and pan‐CK (Abcam, ab7753, 1:100) (Akoya Biosciences) (Table S2, Supporting Information). This was followed by incubation with secondary antibodies and corresponding reactive Opal fluorophores. Nuclei acids were stained with 4′,6‐ diamidino‐2‐phenylindole (DAPI). As negative control, tissue slides were incubated with primary and secondary antibodies, without fluorophores to assess autofluorescence. Multiplex stained slides were scanned using a Vectra Polaris Quantitative Pathology Imaging System (Akoya Biosciences) at 20 nm wavelength intervals from 440 to 780 nm with a fixed exposure time and an absolute magnification of ×200. All scans for each slide were then superimposed to obtain a single image. Multilayer images were imported to inForm v.2.4.8 (Akoya Biosciences) for quantitative image analysis. Tumor parenchyma and stroma were differentiated by Pan‐CK staining. The quantities of various cell populations were expressed as the number of stained cells per square millimeter and as the percentage of positively stained cells in all nucleated cells.

### 2.13 IHC

IHC staining was performed according the standard streptavidin–biotin–peroxidase complex method. Paraffin‐embedded, formalin fixed sections were dewaxed by xylene, rehydrated using graded ethanol, rinsed with deionized water, and then blocked with 3% hydrogen peroxide for 10 min at room temperature. Antigen retrieval was performed by high‐pressure‐cooking the samples in a 10 mm citrate buffer (pH 6.0) for 4 min. Slides were blocked with 5% normal goat serum for 30 min at room temperature and subsequently incubated with primary antibody at 4 °C overnight. The usage information of primary antibodies was listed in Table S2 in the Supporting Information. Slides were then incubated with an Envision detection system (DAKO), and the nucleus was counterstained by Meyer's hematoxylin.

An immunoreactivity score system was applied in the analysis of IHC staining. The percentage of positive cells was scored as follows: 0, <5%, 1, 5–25%, 2, 25–50%, 3, 50–75%, 4, 75–100%. The intensity of staining was scored as follows: 0, negative; 1, weak; 2, moderate; 3, strong. The total score was determined by the following formula: staining index = positive percentage × intensity. The median immunoreactivity score was used as the cut‐point to define cases with high or low expression. To analyze the proportion of *APOE^+^* and *APOC1^+^* cells in tumor, five fields in the representative area for each tumor were imaged considering the tumor heterogeneity. Hematoxylin and eosin staining was performed on serial sections that have been performed with IHC staining to evaluate the boundary of tumor and stroma. The number of staining positive cells in tumor was calculated using the counting tool of Photoshop software.

### 2.14 IF Staining

IF staining was performed on formalin‐fixed, paraffin‐embedded sections. After dewaxed and rehydrated, the slides were incubated with the primary antibodies to SIGLEC1 (#GTX131703, GeneTex; dilution ratio, 1:100); Sialophorin (SPN) (#ab257315, Abcam; dilution ratio, 1:100) (Table S2, Supporting Information) at 4 °C overnight in a moist chamber. After washing thoroughly, Alexa Fluor 594‐ or 488‐ conjugated secondary antibodies (Invitrogen) were used to incubate the slides. At last, all slides were mounted with antifade reagent with DAPI (#S36938, Thermo Fisher Scientific). Images were captured with a ZEISS LSM880 fluorescence microscope.

### 2.15 Statistics Analysis

Two‐sided nonparametric Wilcoxon rank sum test, log‐rank tests, one‐sided permutation test, two‐sided Pearson correlation test, and two‐sided Kruskal–Wallis test were performed with R (version 4.0.2). Kaplan–Meier plots and log‐rank tests were used for overall survival analysis. The unpaired two‐tailed *t*‐tests with Welch's correction were used for two‐group comparisons. Linear regression analysis was applied for correlation test. The results were considered statistically significant if *P* < 0.05.

## 3 Results

### 3.1 Landscapes of Cell Composition in Normal, Primary Tumor, and Lymph Node Metastatic Microenvironment

To better understand the landscapes of microenvironments in ESCC, single‐cell RNA sequencing was applied to analyze cell composition in surgical resected specimens from four ESCC patients, including PT tissue, adjacent nontumor tissue (N) and LNM (three cases) or without metastasis (LN, one case) (Figure 1a and Table S1, Supporting Information). After rigorous quality filtering and doublet removal (see the Experimental Section), a total of 85 263 cells were obtained from these four patients, with the average of about 1400 expressed genes and 4965 UMIs, including 24 372 cells from N, 27 979 cells PT, 24 751 cells from LNM and 8161 cells from LN (Figure 1b and Data S1, Supporting Information), suggesting sufficient coverage and representative of transcripts.

To reduce the dimensionality and group of cells with similar expression profiles, we used principles component analysis and Uniform Manifold Approximation and Projection (UMAP) algorithm implemented in Seurat software^[^ ^11^ ^]^ to identify cell clusters in the TME. Each cluster was annotated as a specific cell type with the expression of well‐known marker genes to form six major cell types, including 18 234 B cells (*CD79A*, *MS41*, *CD79B*, and *CD19*), 40 291 T cells (*CD3D*, *CD2*, *CD3E*, *CD3G* and *CD8A*), 10 081 epithelial cells (*KRT5*, *KRT15*, *KRT18*, *KRT19*, and *EPCAM*), 7857 fibroblast cells (*DCN*, *COL1A1*, and *COL3A1*), 7198 myeloid cells (*LYZ*, *MS4A6A*, and *CD68*) and 1602 endothelial cells (*PECAM1*, *EGFL7*, and *VWF*) (Figure 1b,c). Comparison analysis of the fraction of six major cell types between four distinct tumor ecosystems showed that the abundance change in cell compositions is dynamic, suggesting heterogeneity and complexity of tumor ecosystems in ESCC (Figure 1b,d). To reveal the diversity of major cell types, we performed further subcluster analysis of each major cell type to identify heterogeneous subgroups. 20 subgroups in B cells, 31 subgroups in T cells, 19 subgroups in Epithelial cells, 18 subgroups in Fibroblast cells, 18 subgroups in Myeloid cells, and 8 subgroups in Endothelial cells were successfully defined, most of which were shared by all four microenvironments. Some of subgroups were only found in primary tumor (T‐C19, T‐C21, and Fibroblast‐C9) or lymph node metastasis (B‐C8, B‐C18, T‐C4, Myeloid‐C3, Myeloid‐C4, and Endothelial‐C4) tumor ecosystem (Figure 1e and Data S1, Supporting Information), indicating their special function or potential role in the tumorigenesis and metastasis of ESCC.

### 3.2 T Cell Sub‐Clustering and Their Diverse Enrichment in ESCC Progression

**Figure 2:**
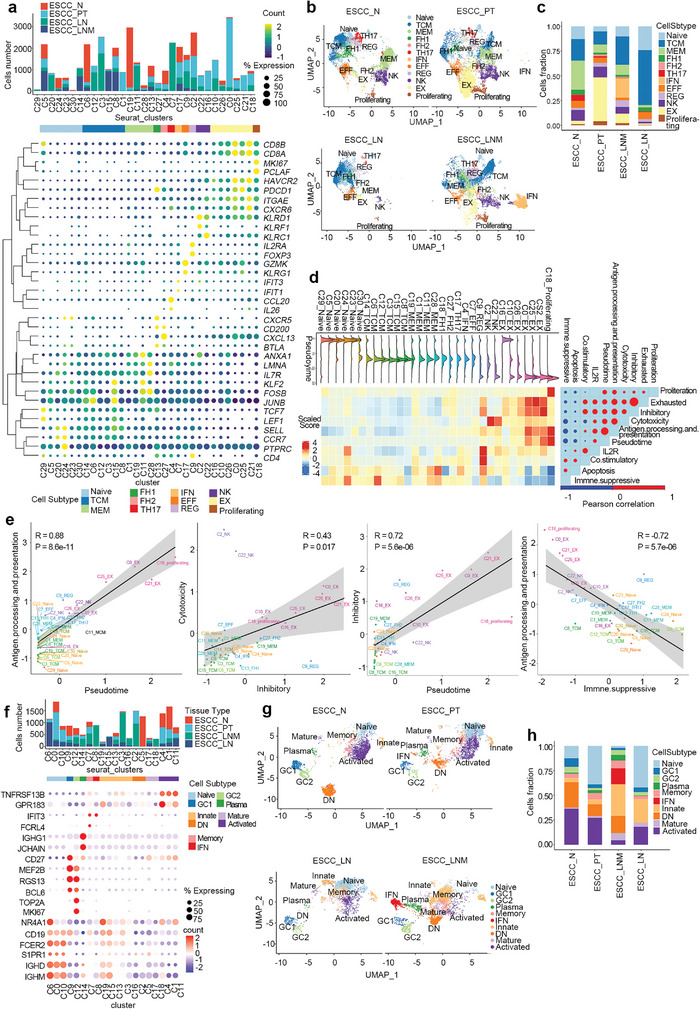
Distinct states of T cells, B cells in ESCC. a) Differential expression of functional state signatures for T cells subclusters and stacked bar plot showing the cell number of T cells with distinct states, color‐coded by their originated ecosystem. Dot size: percent of cells expressing gene and dot color: scaled average expression. b) UMAP plot of annotated subclusters for 40 291 T cells, separated by their distinct originated ecosystems. c) Annotated subclusters fractions relative to the total T cells per ecosystem. Each stacked bar represents an ecosystem for which the total T cell count was scaled to 1. d) Heatmap showing the dynamic changes in known functional signature scaled scores along the pseudotime (upper panel). The distribution of T cells subclusters during the transition, along with the pseudotime (upper panel). Correlation analysis of functional signature scores and pseudotime (right panel). Dot size, the value of Pearson correlation coefficient; dot color, red: positive, blue: negative. e) Significant correlation of scaled scores (antigen processing presentation versus pseudotime, cytotoxicity versus inhibitory, inhibitory versus pseudotime, and antigen processing presentation versus immune suppressive). f) Differential expression of functional state signatures for B cells subclusters and stacked bar plot showing the cell number of B cells with distinct states, color‐coded by their originated ecosystem. g) UMAP plot of annotated subclusters for 18 234 B cells, separated by their distinct originated ecosystems. h) Annotated subclusters fractions relative to the total B cells per ecosystem. Each stacked bar represents an ecosystem for which the total B cell count was scaled to 1.

Landscape view of cell composition showed that T cells were the most dominant component of ESCC tumor microenvironment. To explore the diverse function of T cell subtypes, we analyzed the gene expression of all 40291 T cells and further sub‐clustered them into 31 subgroups, indicating the diversity of T cell population (Data S1, Supporting Information). Based on canonical markers associated with the phenotype or state of T cells, we calculated their average expression and expressed rate of cells among 31 T cell subgroups (**Figure** **2**a). We found that six subclusters, including C29, C5, C20, C24, C23, and C30, all express high levels of naïve T cell markers, including *TCF7*, *LEF1*, *SELL*, *CCR7*, and *PTPRC*, which are defined as naïve T cells (T naive).^[^ ^7^ ^]^ C14, C6, C12, C3, C15, and C8 subcluster shared a few common genes with naïve T cell markers, such as *SELL* and *TCF7*, but express high levels of *JUNB*, *FOSB*, and *KLF2*, suggesting a central memory T‐cell phenotype (TTCM).^[^ ^23^ ^]^ Subclusters C19, C1, C11, and C28 express *ANXA1*, *LMNA*, and *IL7R*, indicating a memory T cell subtype (TMEM). Subclusters C13 and C14 with the high expression of *BTLA*, *CXCL13*, and *CD200* were defined as follicular helper T cells and C13 also shows higher expression of *CXCR5* and *PDCD1* than C14, suggesting different subsets of follicular helper T cells (C13‐TFH1 and C14‐TFH2).^[^ ^24^ ^]^ Subclusters C17 (*CCL20* and *IL26*), C4 (*IFIT1* and *IFIT3*), C7 (*KLRG1* and *GZMK*), C9 (*FOXP3* and *IL2RA*), C2 (*KLRD1* and *KLRF1*), and C22 (*KLRC1*) were defined as T helper 17 cells (C17‐TTH17), interferon‐induced T cells (C4‐TIFN), effector T cells (C7‐TEFF), regulatory T cells (C9‐TREG), and nature killer cells (C2‐TNK and C22‐TNK) according to their differential expression of canonical markers. Interestingly, we found seven subclusters including C16, C10, C26, C0, C25, C21, and C18, expressed high levels of immune checkpoint genes (*HAVCR2* and *PDCD1*), which could be defined as exhausted T cells (TEX). Seven exhausted T cell subsets showed heterogenous expression profile. For example, exhausted T cell subcluster C21 also highly expressed resident memory markers (*ITGAE* and *CXCR6*), while subcluster C18 uniquely expressed *MKI67* and *PCLAF* associated with mitosis and proliferation (C18‐TProliferating), suggesting a diversification of exhausted T cells.

To explore the difference of T cell composition among distinct tumor ecosystem, we displayed the distribution of T cell subtypes in different tumor ecosystem and compared their fraction difference. The results showed that exhausted T cells are enriched in primary tumor ecosystem, whereas naïve/central memory T cells contributed mainly to T cell composition of lymph node without metastasis (Figure 2b,c), suggesting progressive loss of function of effector T cells in primary tumor ecosystem. Intriguingly, interferon‐induced T cells are only existing in metastatic lymph node and their highly expressed *IFIT1*/*IFIT3* genes have been associated with tumor growth, regional, and distant metastases in oral squamous cell carcinoma,^[^ ^25^ ^]^ indicating the unique role of interferon‐induced T cells in promoting ESCC metastasis. While the function of IFNs‐induced immune cells subsets in lymph node metastasis remained unclear. In present study, we performed multiplexed IF staining experiment to preliminary explore whether *IFIT1/IFIT3^+^* T cells play tumor‐promoting or cytotoxic roles in lymph node metastasis. The results demonstrated that *IFIT1/IFIT3^+^* T cells were correlated with accumulation of *FOXP3^+^* Tregs in tumor nest of lymph node metastatic (Figure S4a,b, Supporting Information).To further explore the dynamics of T cells in ESCC, we use Monocle2 to inferr the trajectories of T cells from differential expressed profiles and estimated their relative pseudo‐times along trajectories. We observed the dynamic trajectories of T cells initiated at naïve state and intermediated at memory, helper, regulatory or effector state, and finally terminated in an exhausted state (Figure 2d). Combined with scores for other functional modules of T cells, with the development of T cells, scores for antigen processing presentation (*R* = 0.88, *p* = 8.6 × 10^−11^), inhibitory (*R* = 0.72, *p* = 5.6 × 10^−06^), cytotoxicity, exhaustion, and proliferation increased, while scores of costimulatory, apoptosis, and immune suppressive reduced (Figure 2d,e). A positive correlation of cytotoxicity with inhibitory (*R* = 0.43, *p* = 0.017) and a negative correlation between antigen processing presentation and immune suppressive (*R* = −0.72, *p* = 5.7 × 10^−06^) are observed to be significant (Figure 2e). Using bulk RNA sequencing data from TCGA‐ESCC cohort, we estimated the expression of marker gene signatures for each T cell subtype in TCGA‐ESCC patients using single‐sample gene set enrichment analysis and assessed their prognostic value. The results found that C20_TNaive, C6_TTCM, C7_TEFF, C28_TMEM, C17_TTH17, C27_TFH2, C10_TEX, and C26_TEX was associated with poorer prognosis and C18_TProliferating was correlated with better prognosis, suggesting that their abundance in TME could be used to predict the clinical outcome of ESCC patients (Figure S1a and Data S2 and S3, Supporting Information).

### 3.3 Diversity of B Cells in ESCC

We identified 20 B cells subclusters, which can be categorized into ten functionally distinct subtypes, including Naïve (*IGHD*, *IGHM*, *S1PR1*, *FCER2*, *CD19*, and *NR4A1*), GC1 (geminal center‐1: *CD27*, *MEF2B*, *RGS13*, and *BCL6*), GC2 (geminal center‐2: *MKI67*, *TOP2A*, *RGS13*, and *BCL6*), Plasma (*IGHG1* and *JCHAIN*), Memory (*FCRL4*), IFN (interferon‐induced: *IFIT3*), Innate (*NR4A1*), DN (double negative: *IGHD‐*, *IGHM‐*, and *CD27*‐), Mature (*IGHD‐*, *IGHM‐*, and *CD27*), and Activated (*TNFRSF13B* and *GPR183*) (Figure 2f,g). The fraction of *FCRL4* ^+^ *IFIT3^+^* memory B cells in tumor microenvironment (including primary tumor and lymph node metastasis) is higher than that in the tumor‐free microenvironment (including normal tissue and lymph node) (Figure 2h), and *FCRL4* as a inhibitory receptor expressed in B cells, and expansion of this subtype might result in the poor acquisition of immunity due to loss of response to soluble antigens.^[^ ^26^ ^]^ The innate‐like B cells express moderate levels of dual‐features including naïve and activated markers, with a predominance also found only in lymph nodes with and without tumor metastasis (Figure 2f–h). Interestingly, the interferon‐induced B cells was only found in metastatic lymph node, while interferon‐induced T cells are located at the same site, suggesting an important role of interferon‐induced gene expressions in immune cells in promoting lymph node metastasis of ESCC (Figure 2g,h). Prognostic analysis showed the higher expression of early stage of germinal center B cells (GC1) shows worse prognosis while that of the late stage of germinal center B cells (GC2) was proved to be with better prognosis (Figure S1b, Supporting Information), suggesting that the function of germinal center B cells is dynamic. C4_Activated B cells and three other major lymph node metastasis infiltrated B cell subgroups including C3_Innate, C16_Innate and C2_DN were identified to be associated with worse prognosis (Figure S1b and Data S2 and S3, Supporting Information).

### 3.4 The Accumulation of Metastatic Macrophage Cells in ESCC Lymph Node with Metastasis

**Figure 3:**
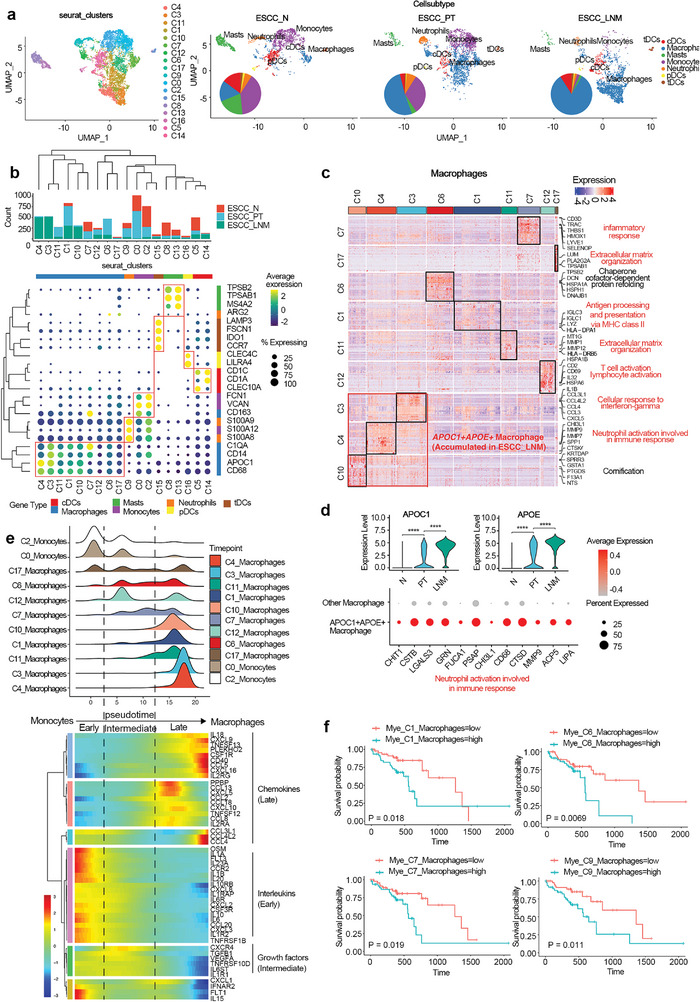
Expression and development of myeloid‐derived cells in ESCC. a) UMAP plot of 7198 myeloid‐derived cells showing the components and relative abundance of cells subtypes, color‐coded by subclusters (left) and annotated cell subtypes (right). b) The expression of marker genes for defining myeloid‐derived cells subtypes. c) Heatmap of genes with differential expression (rows) among the macrophages cells subtypes, highlighted by corresponding markers and enriched pathway of Gene Oncology analysis. d) The identification of metastatic‐specific macrophages characterized by the dual positive expression of *APOC1* and *APOE* genes, which usually involved in neutrophil activation. e) Heatmap showing the dynamic changes in interleukins, growth factors, and chemokines expression (lower panel) along with the pseudotime during the transition of monocytes to macrophages (upper panel). f) Kaplan–Meier curves for overall survival in the 95 patients in TCGA‐ESCC cohort stratified according to high versus low score of the selected marker signature.

A total of 7196 myeloid cells are clustered into 18 subclusters, including nine macrophages, one neutrophils, two monocytes, two mast cells, one tolerogenic dendritic cells, one plasmacytoid dendritic cells, and two conventional dendritic cells (**Figure** **3**a,b). More than half of myeloid cells are found to be derived from monocytes and macrophages. Interestingly, the composition of myeloid‐derived cells changed dramatically from normal to tumor and metastatic lymph node microenvironments (Figure 3a). For example, the fraction of monocytes decreased from 39.28% in normal tissue to 31.74% in primary tumor and <1% in metastatic lymph node. In contrast, the fraction of macrophage increased from 16.41% in normal tissue to 51.57% in primary tumor and 82.6% in metastatic lymph node (Figure 3a), implying that the myeloid‐derived cells were remodeled in TME and the potential transition of monocytes to macrophages may play critical roles in ESCC development and progression. Increased macrophages have nine subtypes expressing different transcriptomic files, including the enrichment of inflammatory responses in the C7 subgroup, extracellular matrix (ECM) organization in C17 and C11 subgroups, chaperone cofactor‐dependent protein refolding in C6 subgroup, antigen processing and presentation via major histocompatibility complex (MHC) class II in C1 subgroup and T cell activation in C12 subgroup. More interestingly, macrophage subgroups C3, C4, and C10 are uniquely existed in LNM microenvironment, which can be defined by the expression of *APOC1* and *APOE*. In addition, genes in the interferon‐mediated signaling pathway as well as genes related to neutrophil activation are also upregulated in these metastatic macrophages (Figure 3c,d). Macrophages secreted variety of cytokines at different stages, including interleukins (*IL1A*, *IL1B*, *IL23A*, *IL20*, *IL10*, *IL6*, and *IL15*) for regulating immune responses and inflammatory reactions at the early stage of the tumor, followed by growth factors (*CXCR4*, *TGFB1*, *VEGFA*, and *TNFRSF10D*) for supporting tumor growth, and finally chemokines (*CXCL5*, *CXCL9*, *CXCL10*, *CXCL16*, *CCL2*, *CCL4*, *CCL5*, *CCL8*, and *CCL18*) for promoting tumor cell migration and invasion by inducing epithelial‐mesenchymal transition (Figure 3e). Three macrophage subtypes (C1, C6, and C7) and neutrophils are also defined as prognostic myeloid cells associated with poorer clinical outcomes (Figure 3f and Data S2 and S3, Supporting Information). These results demonstrated that metastatic tumor microenvironment could be characterized by the high density of tumor associated macrophages, which promoted tumor invasion and metastasis through secreted chemokines. These prometastatic macrophages could be therapeutic targets to prevent ESCC progression by interfering with their accumulation in the metastatic TME.

### 3.5 Cancer Associated Fibroblasts (CAFs) and Pericytes Associated with Metastasis in ESCC

**Figure 4:**
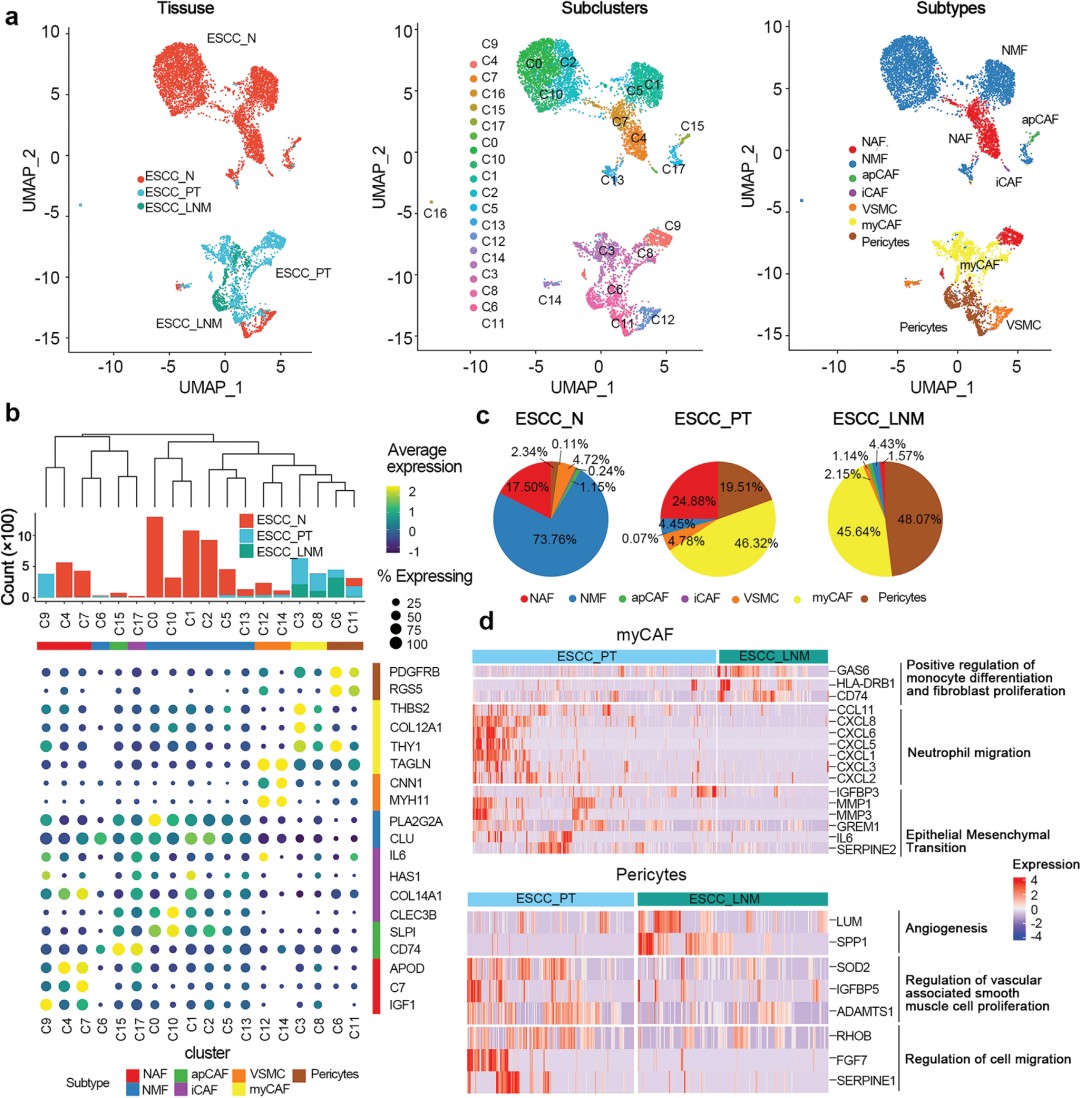
Fibroblast cells subtypes in ESCC. a) UMAP plot of 7857 Fibroblast cells showing the components of cells subtypes, color‐coded by originated ecosystem (left), subclusters (middle), and annotated subtypes (right). b) The identification of fibroblast cell subtypes. c) The accumulation of myCAFs and Pericytes in primary tumor and metastatic lymph node ecosystem. d) Heatmap showing the differential expression of genes between primary tumor and lymph node with metastasis for myCAFs and Pericytes.

We also identified 18 subclusters with exclusively expressed markers for 7857 fibroblast cells, three for normal activated fibroblasts (NAF), seven for normal mucosa fibroblasts (NMF), two for vascular smooth muscle cells, two for pericytes, and four for CAF (**Figure** **4**a,b and Figure S2, Supporting Information). Based on the differential expression of canonic CAF markers, four CAF subsets could be defined as one antigen presenting CAF (apCAF: *CD74*, *HLA‐DRA*, and *HLA‐DPB1*), one inflammatory CAF (iCAF: *IL6*, *HAS1*, *COL14A1*), and two myofibroblasts (myCAF: *THBS2*, *COL12A1*, and *THY1*) including C8_CAF with highly expressed immunoglobulin genes (*IGKC*) and C3_CAF with expression of ECM component and matrix metallopeptidase genes (*POSTN*, *COL1A1*, *CTHRC1*, *MMP1*, and *MMP11*), which could bind to integrins to support adhesion and migration of epithelial cells as reported (Figure 4a,b and Figure S2, Supporting Information).^[^ ^22^, ^23^ ^]^ Intriguingly, the myCAFs are uniquely existed and consisted of nearly 50% fibroblasts in primary tumor and metastatic lymph node TMEs, which are clearly separated from the normal fibroblasts (NAF and NMF) in normal tissue (Figure 4a,c). Further analysis revealed distinct expression features of myCAFs at different stages of ESCC development. In primary tumors, myCAFs secrete multiple cytokines (*CCL11*, *CXCL1*, *CXCL2*, *CXCL3*, *CXCL5*, *CXCL6*, *CXCL6*, and *CXCL8*) for neutrophil recruitment and upregulate genes for inducing epithelial‐mesenchymal transition (*IL6*, *SERPINE2*, *GREM1*, *MMP1*, and *MMP3*). In metastatic lymph nodes, myCAFs are characterized by upregulating genes to promote monocyte fibroblast proliferation (Figure 4d). These results suggest the heterogeneity of fibroblasts and the potential role of high‐density myofibroblasts in tumor progression and metastasis. Pericytes are distributed from 2.34% of fibroblasts in normal tissue to 19.51% of primary tumor fibroblasts with the upregulation of genes related to cell migration and proliferation of vascular associated smooth muscle cell, and to 48.07% of fibroblasts at metastatic lymph nodes characterized by the activation of angiogenesis related genes (*LUM* and *SPP1*), suggesting that the accumulation of pericytes might promote the tumor growth and metastasis (Figure 4c,d). In these subsets, C3_myCAF, C8_myCAF and C6_pericyte were uniquely discovered in tumor and metastatic lymph node. Interestingly, C6_ pericyte mostly enriched in metastatic lymph node with highly expressed *THY1, PDGFRβ, RGS5*, and *NDUFA4L2*. Previous studies have reported two main mechanisms of pericytes for promoting tumor growth and metastasis, one is the pericyte‐CAF transition induced by PDGF‐BB‐PDGFR*β* signaling,^[^ ^27^ ^]^ which is also consistent with our observations in the present study that *PDGFRB* is upregulated in pericytes and similarity between pericytes and CAFs in UMAP clustering plot (Figure 4a,b). Another one is aberrant tumor angiogenesis caused by the high amount of pericytes coverage on tumor vessels, which improves tumor vessel stability and perfusion, thereby ensuring the delivery of nutrients to support tumor growth and preventing tumor cell dissemination through blood vessels.^[^ ^28^, ^29^, ^30^ ^]^ In addition, *THY1* was found functioning as both cancer stem cell and CAF marker in ESCC in our previous study.^[^ ^31^ ^]^ Thus, *THY1^+^PDGFRβ^+^* pericyte maybe function as a subset of CAF possessed pluripotent potential in promoting ESCC lymph node metastasis. Notably, two hypoxic induced factors *RGS5* and *NDUFA4L2* were highly expressed in this subset. In all these markers, *THY1^+^NDUFA4L2^+^* pericytes were validated to be significantly increased in metastatic lymph node than primary tumor by multiplexed IF staining (Figure S4c,d, Supporting Information). However, the detailed mechanism of *THY1^+^NDUFA4L2^+^* pericytes in ESCC lymph node metastasis need further study.

### 3.6 The Heterogeneity of Epithelial Cells

**Figure 5:**
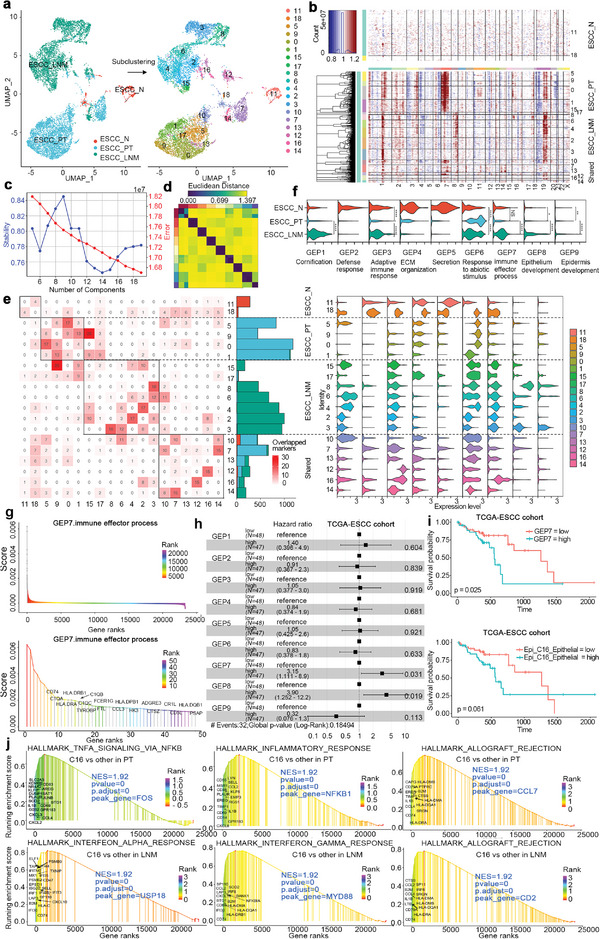
Heterogeneity of malignant epithelial cells in ESCC. a) UMAP plot of 10 081 epithelial cells, color‐coded by originated ecosystem (left) and subclusters (right). b) Heatmap showed the large‐scale CNVs for epithelial cells (rows along y‐axis) from normal adjacent, primary tumor, and metastatic lymph node ecosystem. Red: amplification and blue: deletion. Epithelial cells from different subclusters and the range of different chromosomes are labeled by different color bars on the left and top to the heatmap, respectively. c) The stability and error curve of inferred gene expression program (GEP) numbers with the range of from 5 to 19, implemented by consensus non‐negative matrix factorization (cNMF) algorithm. The optimal number is 9. d) Heatmap showing the Euclidean distance of programs across replicates. e) Heatmap (right) showing the number of overlapped marker genes across 19 Epithelial cells subclusters. Stacked bar plot (left) showing the cell number of subclusters, color‐coded by their originated ecosystem. f) The violin plot of expression level of 9 gene expression programs (GEPs) across distinct ecosystems (upper) and subclusters (lower). g) The contributed score of all genes (upper) and top50 genes (lower) for GEP7 immune effector process, ranked and color‐coded by their contributed score. h) Prognostic values of nine GEPs in the 95 patients in TCGA‐ESCC cohort. Forest plots show Hazard Rations and horizontal ranges derived from Cox regression survival analyses for overall survival. Significant results are indicated with *. i) Kaplan–Meier curves for overall survival in the 95 patients in TCGA‐ESCC cohort stratified according to high versus low score of the GEP7 and C16_Epithelial signatures. j) Enriched tumor hallmark pathway of differentially expressed genes in C16_epithelial cells comparing with other epithelial subclusters in primary tumor (upper) and lymph node with metastasis (lower).

To explore the heterogeneity of 10 081 epithelia cells, we analyzed their transcriptomic profile and performed clustering analysis. The results showed that epithelial cells can be divided into three groups based on their tissue‐specific expression profiles, including normal epithelial cells (ESCC_N), primary tumor cells (ESCC_PT), and lymph node metastatic tumor cells (ESCC_LNM). Same group cells also existed diversity and could be clustered into 19 subclusters (**Figure** **5**a), which were validated by the chromosomal copy number variation profile analysis (Figure 5b). Compared to the reference data of normal epithelial cells (ESCC_N: C11, C18), the primary and lymph node metastatic malignant epithelial cells presented a large‐scale diverse in chromosomal copy number alteration patterns. For example, amplification of chromosome 1 and deletion of 19 in specific regions could be frequently detected in both ESCC_PT and ESCC_LNM, whiles amplifications of chromosomes 7 and 8 were only observed in ESCC_PT and ESCC_LNM, respectively (Figure 5b). Top 50 significantly differentially expressed genes for each subcluster ranked by fold changes were identified, and their low interoverlapped number between subclusters also demonstrated the high heterogeneity of epithelial cells (Figure 5e). To further explore the heterogeneous function hidden by gene expression profile, we applied non‐negative factorized matrix algorithm on the total gene expression count matrix of all epithelial cells to extract gene expression programs of epithelial cells. After numerous permutation tests and the trade‐off evaluations of stability and errors, 9 GEPs are identified and exhibited distinct expression level among 19 subclusters or 3 subgroups (Figure 5c,d,f and Data S4 and S5, Supporting Information). Normal epithelial cells express higher level of homeostasis maintenance‐related programs including GEP2, GEP4, and GEP5. Malignant epithelial cells expressed more keratinization (GEP1, GEP9), epidermal cell differentiation (GEP1, GEP9) and T cells activation (GEP3) related programs, while the lymph node metastatic epithelial cells expressed extremely high levels of immune effector process regulation (GEP7) and O‐glycan processing (GEP8) (Figure 5f,g and Figure S3, Supporting Information), which are identified as predicting poorer clinical outcome in the multivariate cox regression analysis of nine GEPs based on TCGA‐ESCC cohort (hazard ratio (HR) = 3.15, *P* = 0.031; HR = 3.9, *P* = 0.019) (Figure 5h). Interestingly, C16 subcluster with the highest score of regulating immune effector process highly express *CD74*, *CXCR4*, and HLA‐class II related genes (Figure 5g), and also tend to predict worse clinical outcome (*P* = 0.061 and Data S2 and S3, Supporting Information) (Figure 5i). Gene set enrichment analysis (GSEA) analysis of differentially expressed genes between C16 and other subclusters revealed that tumor necrosis factor alpha (TNFA)‐signaling via nuclear factor kappa‐B (NFKB) and inflammatory response pathway is enriched in primary malignant C16 epithelial cells while the interferon response pathway was enriched in lymph node metastasis C16 epithelial cells (Figure 5j), consistent with previous reports that the CD74‐MIF‐CXCR4 axis is involved in inflammatory and atherogenic immune cell recruitment, stem cell homing, and cancer cell metastasis.^[^ ^32^, ^33^, ^34^ ^]^

### 3.7 Differential Intercellular Communications of Diverse Tumor Ecosystems

**Figure 6:**
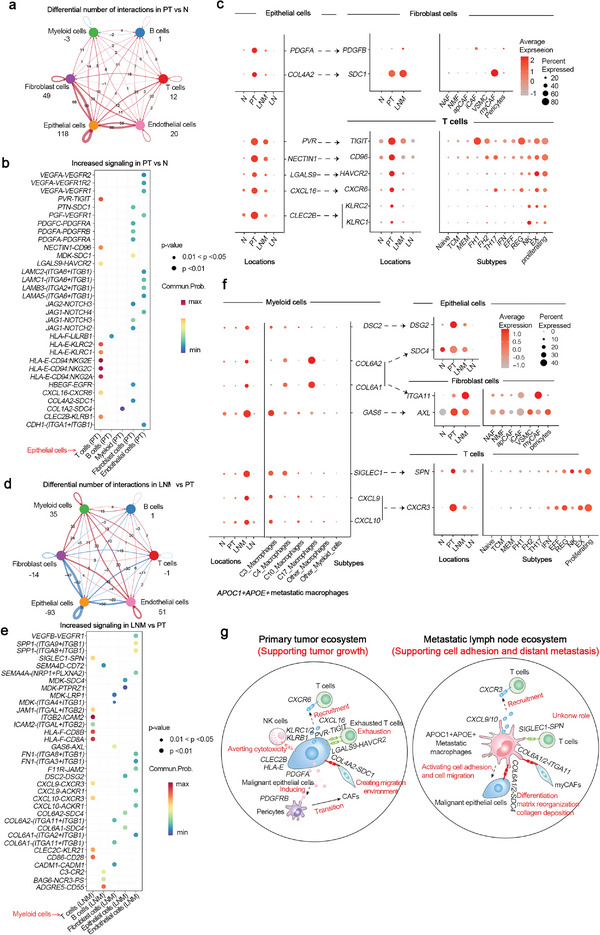
Different tumor immune phenotypes between primary tumor and metastatic lymph node shaped by crosscompartment interactions. a) Circle plot shows the difference of inferred intercellular communication network for primary tumor versus normal adjacent in ESCC, where red colored edges represent increased signaling while blue colored edges represent decreased signaling (labeled by number). b) The upregulated signaling ligand–receptor pairs between epithelial cells and other compartments in primary tumor compared to normal adjacent. Dot sizes represent *P*‐value (only *P* < 0.05 shown). c) Dot plot of the differential expression of significantly upregulated signaling ligand–receptor pairs by compartment and cell types in primary tumor ecosystem. d) The difference of inferred intercellular communication network in metastatic lymph node versus primary tumor. e) The increased signaling ligand–receptor pairs between macrophages cells and other compartments in metastatic lymph node. f) The increased expression of significantly upregulated signaling ligand–receptor pairs in macrophages. g) Proposed model of the crosscompartment signaling ligand–receptor interactions in the context of primary tumor and metastatic lymph node ecosystem.

To explore the mechanism of the accumulation of IFN‐induced immune cells, metastatic macrophages, pericytes, and cancer associated fibroblasts in lymph node metastasis, we analyzed the differential intercellular communications of normal, primary tumor, and lymph node metastasis ecosystems. Compared with normal ecosystem, the number and intensity of proteins in tumor cells interacted with T cells and fibroblasts obviously increased in primary tumor ecosystem (**Figure** **6**a). For examples, upregulation of *CLEC2B* and *HLA‐E* in tumor cells can bind to *KLRB1* or *CD94/NKG2* on the surface of nature killer cells, TH17 and memory T cells to avert natural killer (NK) cell‐mediated tumor elimination.^[^ ^35^, ^36^ ^]^ Expressions of LGALS9, NECTIN1, and poliovirus receptor (PVR) in tumor cells can promote T cells exhaustion by binding to inhibitory receptors of T cells HAVCR2, CD96, and T‐cell immunoreceptor with Ig and ITIM domains (TIGIT), respectively. Expressions of COL4A2 in tumor cells can establish a migration and invasion‐permissive microenvironment by binding to SDC1‐postive myofibroblasts^[^ ^37^ ^]^ and induce the transition of PDGFRB‐positive pericytes to cancer associated fibroblasts by secreting platelet‐derived growth factor subunit A (PDGFA) (Figure 6b).^[^ ^27^ ^]^ These interactions are also supported by the exclusive expression of above ligand‐receptors from epithelial cells‐T cells and epithelial‐fibroblasts (Figure 6c). Compared with primary tumor ecosystem, the lymph node metastatic microenvironment strengthens the crosstalk of myeloid cells with T cells, fibroblasts and epithelial cells (Figure 6d). CXCL9, CXCL10, and SIGLEC1 uniquely secreted by C17 myeloid‐derived macrophage and C3 metastatic macrophages (APOC1^+^APOE^+^ macrophages) can bind to CXCR3 or SPN on the surface of T cells, thereby regulating multiple T‐cell functions, including T‐cell activation, proliferation, differentiation, trafficking and migration (Figure 6e,f),^[^ ^38^, ^39^, ^40^, ^41^ ^]^ but their roles in ESCC metastasis remained unclear. APOC1^+^APOE^+^ metastatic macrophages also express COL6A1 and COL6A2, which are independent predictors of poor prognosis and indicators of distant metastasis in pancreatic cancer,^[^ ^38^ ^]^ are associated with myofibroblast differentiation, matrix reorganization, and collagen deposition in breast cancer by interacting with myofibroblast cells via ITGA11.^[^ ^42^ ^]^ In addition, COL6A1 and COL6A2 can interact with epithelial cells via SDC4 to activate cell adhesion and migration (Figure 6e,f). These results indicated that the intercellular communications of primary and metastatic TME are dynamic and mainly rely on the crosstalk of several specific cell types with important function (C17, C3, and C4 APOC1^+^APOE^+^ metastatic macrophage cells enriched in metastasis, T cells with proliferation or exhaustion, PDGFRB^+^ pericytes and C3 POSTN^+^ myofibroblasts accumulated in metastasis). Detailed effects and underlying mechanisms of these ligand‐receptor interactions on ESCC progression should be further characterized, which may lead to identify novel therapeutic targets to enhance immunotherapy efficiency or inhibit tumor growth and metastasis.

### 3.8 APOC1^+^APOE^+^ Macrophages Mediate the Establishment of Immune Microenvironment of Lymph Node Metastasis in ESCC

**Figure 7:**
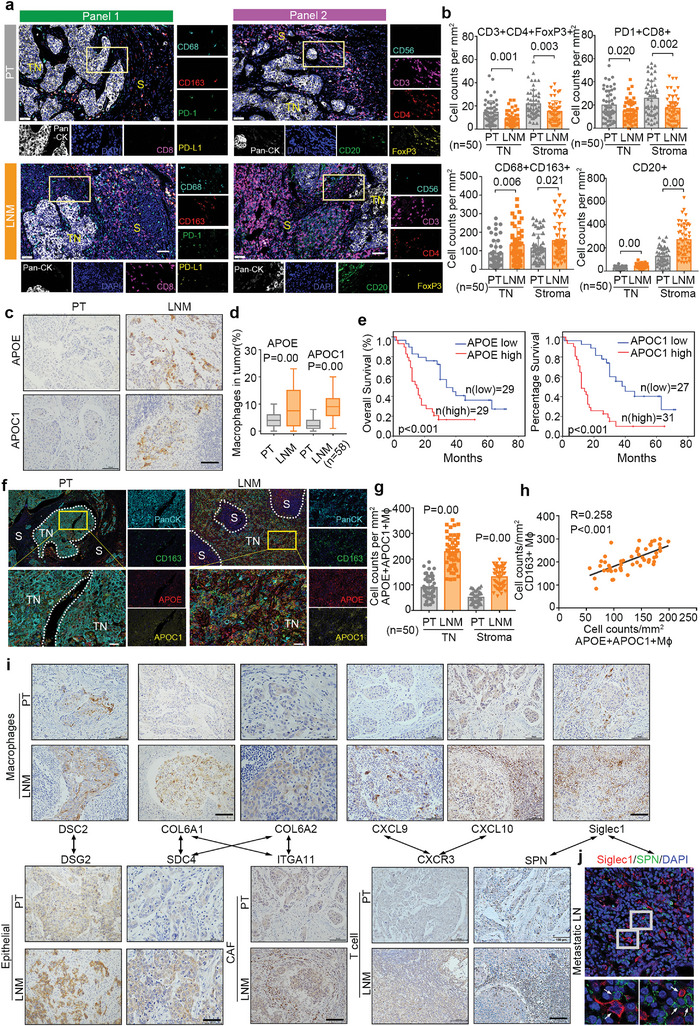
*APOC1+APOE+* macrophages mediate the establishment of immune microenvironment of lymph node metastasis in ESCC. a) Representative images of multiplexed IF staining of tumor and metastatic lymph node samples using Panel‐1 and Panel‐2. Scale bar, 50 µm. b) Cell density of CD3^+^CD4^+^FoxP3^+^ Treg cells, exhaustive CD8^+^PD1^+^ T cells, CD68^+^CD163^+^ macrophages, CD20^+^ B cells in both tumor nest, and stroma between primary tumor and metastatic lymph node (*n* = 50). c) Representative images of IHC staining of APOE and APOC1 in metastatic lymph node and primary tumor. Scale bar, 100 µm. d) Cell percentage of APOE^+^APOC1^+^ macrophages in in tumor and metastatic lymph node (*n* = 58). e) Kaplan–Meier curve of overall survival of ESCC patients stratified by the abundance of APOE and APOC1 in an independent cohort. f) Spatial characterization of APOE^+^APOC1^+^ macrophages in tumor and metastatic lymph node by multiplexed IF staining. Scale bar, 50 µm. g) Cell density of APOE^+^APOC1^+^ macrophage in both tumor nest and stroma between primary tumor and metastatic lymph node. h) Correlation between CD163+ macrophages and APOE^+^APOC1^+^ macrophages. i) Representative images of IHC staining of DSG2, DSG2, SDC4, COL6A1, COL6A2, CXCL9, CXCL10, CXCR3, ITGA11, Siglec1, and SPN in metastatic lymph‐node and primary tumor (*n* = 20). Scale bar, 100 µm. j) Coexpression of Siglec1 (red) with SPN (green) in metastatic lymph node were shown by double IF staining. Cell nuclei were stained with DAPI (blue). The white arrows indicate the location of Siglec1 and SPN positive cells. Scale bar, 50 µm. Data are presented as means ± SD (standard deviation). TN: tumor nest, PT: primary tumor, LNM: metastatic lymph node, Mɸ: macrophages.

The distinction of immune microenvironment between primary tumor and metastatic lymph node were characterized by multiplexed immunofluorescence staining. Two panels of combinations of antibodies for multiplexed IF assays were used: Panel‐1 consisted of CD8+ PD1 (for exhaustive CD8^+^ T cells), CD68+CD163 (for M2‐ like macrophages), and panCK (for epithelial cells); Panel‐2 consisted of CD3+ CD4 *+* FOXP3 (for Treg cells) CD56 (for NK cells), CD20 (for B cells), and panCK (for epithelial cells) (**Figure** **7**a). These multiplexed IF staining were performed on five matched primary tumor and metastatic lymph node from ESCC patients, qualifying an average of ten regions per sample. Using these data, we validated that primary tumors have more CD3^+^CD4^+^FoxP3^+^ Treg cells, PD1^+^CD8^+^ exhaustive T cells than metastatic lymph nodes, whereas exhaustive CD68^+^CD163^+^ M2‐like macrophages T cells, CD20^+^ B cells increase in metastatic lymph nodes compared with primary tumors (Figure 7b). Accordingly, we believe that macrophages play an important role in the establishment of the metastatic immune microenvironment in metastatic lymph nodes compared with primary tumor.

In this study, a novel subset of *APOC1^+^APOE^+^* macrophage was identified in metastatic lymph node uniquely. The expression level and prognostic value of *APOC1^+^APOE^+^* macrophages were assessed by IHC staining in 58 cases of ESCC patients with metastatic lymph node. A significantly higher percentage of *APOC1^+^* and *APOE^+^* macrophages was verified in metastatic lymph node than primary tumor (*P* = 0.00) (Figure 7c,d). Kaplan–Meier curves showed that higher proportion of *APOE^+^* and *APOC1^+^* macrophages in metastatic lymph node was correlated with shorter overall survival (Figure 7e). In addition, the spatial characterization of *APOE^+^ APOC1^+^* macrophages in microenvironment of primary tumor and metastatic lymph node were evaluated by multiplex IF staining (Figure 7f). Compared with primary tumor, higher abundance of *APOC1^+^APOE^+^* macrophages were observed in tumor nest of metastatic lymph node (Figure 7g). Besides, significantly positive correlation between *APOC1+APOE+* macrophages and CD163+ macrophages were analyzed in tumor nest (Figure 7h).

Furthermore, based on the crosstalk analysis in this study, we identified that *APOC1^+^APOE* ^+^ macrophages were involved in the establishing of the metastatic immune microenvironment by interacting with tumor and stromal cell. The potential function of the *APOC1^+^APOE* ^+^ metastatic macrophage was preliminary validated by IHC and IF staining. We selected ten cases of ESCC patients with *APOC1+APOE+* and *APOC1‐APOE‐* metastatic lymph nodes respectively. The result of IHC staining confirmed that higher expression of *DSC2*, *SIGLEC1, CXCL9, CXCL10, COL6A1*, *and COL6A2* was observed in *APOC1+APOE+* macrophages. Meanwhile, *DSG2/SDC4*+ epithelial, *ITGA11*+ CAF and *CXCR3+* T cells were verified accumulated in *APOC1^+^APOE^+^* metastatic lymph node. The physical interaction between *SIGLEC1+* macrophage and *SPN+* T cell possibly in metastatic lymph node were proved by IF staining (Figure 7i,j and Figure S4e,f, Supporting Information). These data indicated that *APOC1^+^APOE^+^* macrophages assisted tumor colonization in lymph nodes through matrix reorganization, collagen deposition, and metabolism reprogramming via COL6A1/COL6A2‐SDC4 and DSC2‐DSG2 axis. CXCL9/CXCL10 from metastatic macrophage then regulate T cell trafficking, migration and Treg accumulation by recruiting CXCR3+ T cells. The directly interaction between *SIGLEC1+* macrophage and SPN+ T cell possibly expedited tumor cell lymph node metastasis. However, the underlying mechanism of *APOC1+APOE*+ macrophages in ESCC lymph node metastasis remained unclear and future work is warranted to explore this.

## 4 Conclusion

A deeper understanding of the tumor and its microenvironment will help us to design effective approaches for ESCC treatment. Although TME of primary ESCC has been described in several reports,^[^ ^5^, ^6^, ^7^, ^8^, ^9^ ^]^ the TME of lymph node metastases in ESCC remains unclear. Our study not only provides a high‐resolution landscape of the tumor, immune, and stromal compartments in metastatic lymph node but also highlights metastatic‐specific patterns comparing with primary tumor. In the present study, we found that T cells in primary tumor ecosystem highly express immune checkpoint genes (*HAVCR2* and *PDCD1*) and achieve relatively high exhaustion scores, which is consistent with previous study reporting that exhausted CD8 T cells are major proliferative cell components in the primary TME of ESCC. Pseudo‐time analysis of T cells in our study reveals that T cell developmental trajectories begin in a naïve state and end in an exhausted state, consistent with previous understanding. Furthermore, upregulation of PDGFRB in pericytes and a tight topological space between pericytes and CAFs are observed in our study, which supports the pericyte‐CAF transition induced by PDGF‐BB‐PDGFR*β* signaling reported in previous study.^[^ ^27^ ^]^

In this study, we investigated a poor‐prognostic epithelia‐immune dual expression program in ESCC tumor cells characterized by overexpression of immune cells signaling mediators (MHC class II surface receptor CD74, HLA‐DR, and TYROBP), epithelial‐mesenchymal transition related genes (Glucose metabolism regulator *HK3*, Iron metabolism regulator *FTL*), and cell adhesion and migration related gene (*C1Q*, *ADGRE3*, *CTSZ*, and *PSAP*). Similarly, MHC‐II can also express on a subset of cancer associated fibroblast cells (apCAF) to induce naive CD4+ T cells into regulatory T cells (Tregs) in an antigen‐specific manner in pancreatic cancer and express on epithelia cells for contributing to tumor progression in human papilloma virus (HPV)‐related carcinoma mouse model and nasopharyngeal carcinoma (NPC).^[^ ^43^, ^44^, ^45^, ^46^ ^]^ MHC‐II genes have been associated with favorable outcomes in many cancers and usually express on professional antigen‐presenting cells (e.g., macrophages and dendritic cells) to activate CD4^+^ T cells and further support antitumor immunity of CD8^+^ T cell.^[^ ^47^, ^48^, ^49^ ^]^ The role of MHC‐II expression in cancer seems contradictory, but tumor‐specific MHC‐II expression in epithelial‐immune dual expression program indicates the reshape of immune microenvironment by tumor cells and is worth being further studied in the future.

ESCC lymph node metastatic microenvironment picture shows that the emergence or expansion of interferon‐induced T/B cells, POSTN^+^ myofibroblast cells, APOC1^+^APOE^+^ macrophages and intercellular communications through ligand‐receptor to establish premetastatic niche and facilitate metastasis in a profoundly immunosuppressed TME. The global upregulation of interferon responses has been observed in primary TME of NPC, including T cells, B cells, and tumor cells, suggesting the effects of IFNs in the dysfunctional immune states associated with tumorigenesis.^[^ ^45^, ^50^ ^]^ Growing evidence has showed that IFNs may trigger immunosuppressive mechanisms,^[^ ^51^ ^]^ or be cytotoxic, and that their function depends on their concentration in the TME.^[^ ^52^, ^53^ ^]^ Our study is the first to identify IFNs‐induced T cell and B cell subsets that are uniquely existed in lymph node metastatic microenvironment of ESCC. Similarly with a recent study,^[^ ^54^ ^]^ IFIT1+ neutrophils showed distinct PDL1 expression in tumor microenvironment of liver cancer and might suggested immune suppression. Our study firstly demonstrated the IFIT1/IFIT3+ T cells mediated immunosuppression by recruiting FoxP3+ Tregs in metastatic lymph node. Although the detailed function of IFNs‐induced immune cells subsets in lymph node metastasis remained unclear. Our study provides a good starting point for further investigation.

The heterogeneous function of cancer‐associated fibroblasts and tumor‐associated macrophages in tumor initiation, progression, and metastasis have been widely studied in primary TME of multiple cancer types (e.g., prostrate, pancreatic, breast, melanoma, lung, and pancreatic cancers),^[^ ^55^, ^56^, ^57^, ^58^, ^59^, ^60^, ^61^ ^]^ but remains unclear in lymph node metastatic microenvironment. Some recent studies have reported the expression of these genes (e.g., *POSTN*, *APOE*) in subsets of ESCC fibroblasts and macrophages,^[^ ^5^, ^8^, ^9^, ^62^ ^]^ suggesting the existence of these subsets in ESCC microenvironment, but their role in how they interact with cancer cells or immune cells are largely unexplored. In this study, we not only furtherly revealed that POSTN+ myofibroblast cells and APOC1+APOE+ macrophages are major components of the ESCC metastatic microenvironment but also for the first time propose two models of potential intercellular interactions in the context of ESCC tumor ecosystem: one is tumor cells‐led supporting tumor growth in primary tumor and the other is APOC1^+^APOE^+^ macrophages‐mediated supporting cell adhesion and distant metastasis in lymph nodes. The insights provided by these findings may help to shape the TME and inform different therapeutic strategies for inhibiting tumor growth and overcoming metastasis.

## Conflict of Interest

The authors declare no conflict of interest.

## Author Contributions

Y.J. and B.Z. contributed equally to this work. Y.J. performed experiments. B.Z. conceived the study, processed raw sequencing data, and performed all bioinformatics analysis. Y.J. and B.Z. wrote and revised the paper. Y.J., C.Z., Z.C., Z.W., and Y.Q. supervised the sample collection and performed the pathological examination. D.K., S.L., H.H., J.L., Y.Z., X.S., and L.F. participated in discussions. X.G. and Y.Q. supervised the study. All of the authors have read and approved the paper.

## Supporting information

Supporting Information

Supporting Information

## Data Availability

The data that support the findings of this study are available from the corresponding author upon reasonable request.
