# Supplementary material for: Single‐Cell Transcriptomic Analysis of Primary and Metastatic Tumor Ecosystems in Esophageal Squamous Cell Carcinoma
Source: Adv Sci (Weinh). 2023 Jan 29;10(7):2204565. doi: 10.1002/advs.202204565 (PMC9982558; doi:10.1002/advs.202204565)
Supplement: Supplementary file 1 — Supporting Information [file ADVS-10-2204565-s001.pdf]

ADVANCED SCIENCE
Open Access

Supporting Information

for *Adv. Sci.*, DOI 10.1002/advs.202204565

Single-Cell Transcriptomic Analysis of Primary and Metastatic Tumor Ecosystems in Esophageal Squamous Cell Carcinoma

Yongxu Jia, Baifeng Zhang, Chunyang Zhang, Dora Lai-Wan Kwong, Zhiwei Chang, Shanshan Li, Zehua Wang, Huiqiong Han, Jing Li, Yali Zhong, Xin Sui, Li Fu, Xinyuan Guan and Yanru Qin**

Supporting Information

Single-Cell Transcriptomic Analysis of Primary and Metastatic Tumor Ecosystems in Esophageal Squamous Cell Carcinoma

Yongxu Jia#, Baifeng Zhang#, Chunyang Zhang, Dora Lai-Wan Kwong, Zhiwei Chang, Shanshan Li, Zehua Wang, Huiqiong Han, Jing Li, Yali Zhong, Xin Sui, Li Fu, Xinyuan Guan, Yanru Qin**

#These authors contributed equally

**Corresponding authors.*

Supplementary Figure 1

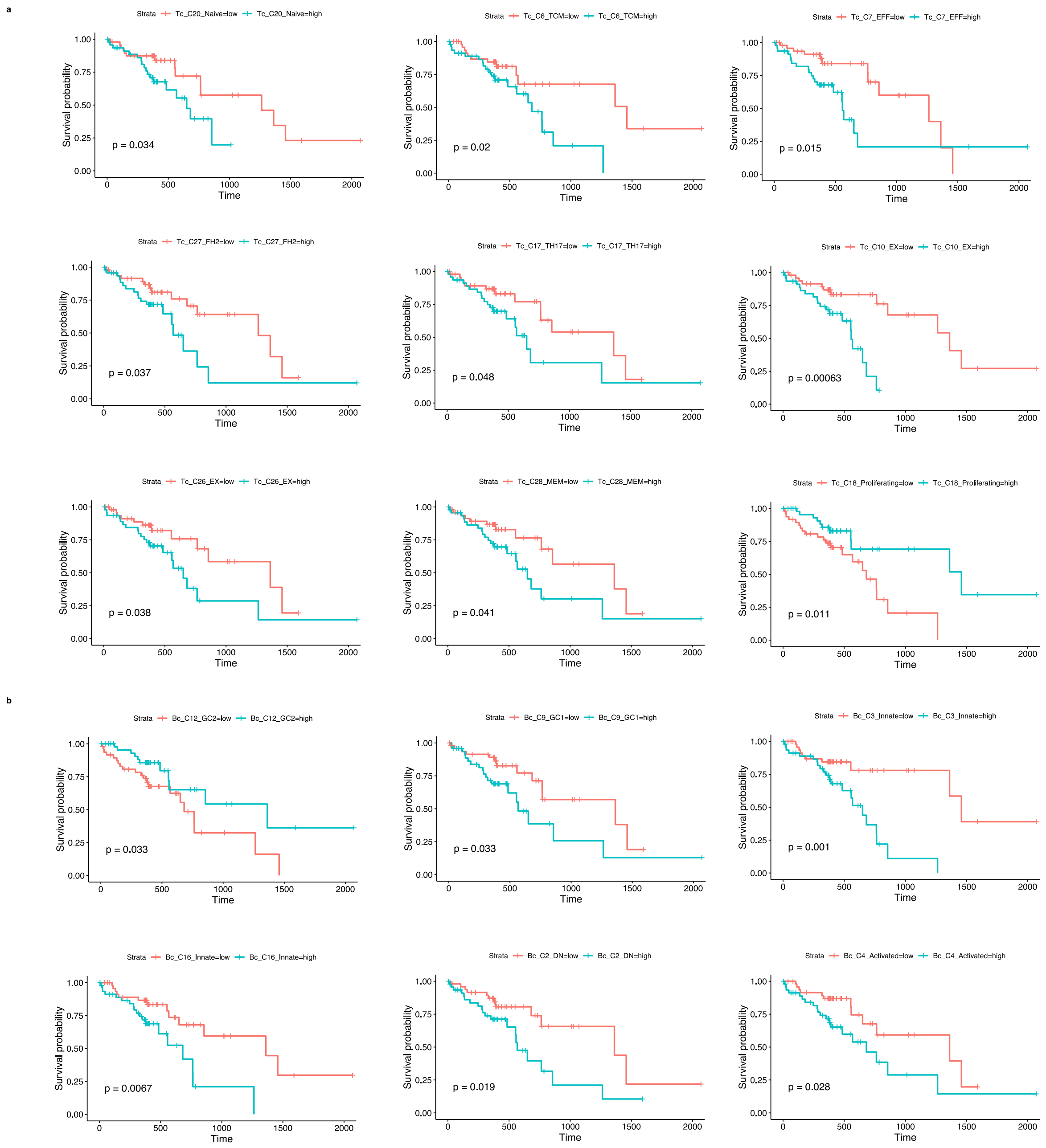

Figure S1. Kaplan-Meier curves for overall survival in the 95 patients in TCGA-ESCC cohort stratified according to high vs low score of the marker signature for each subclusters in T (a), B cells (b). (Only significant shown)

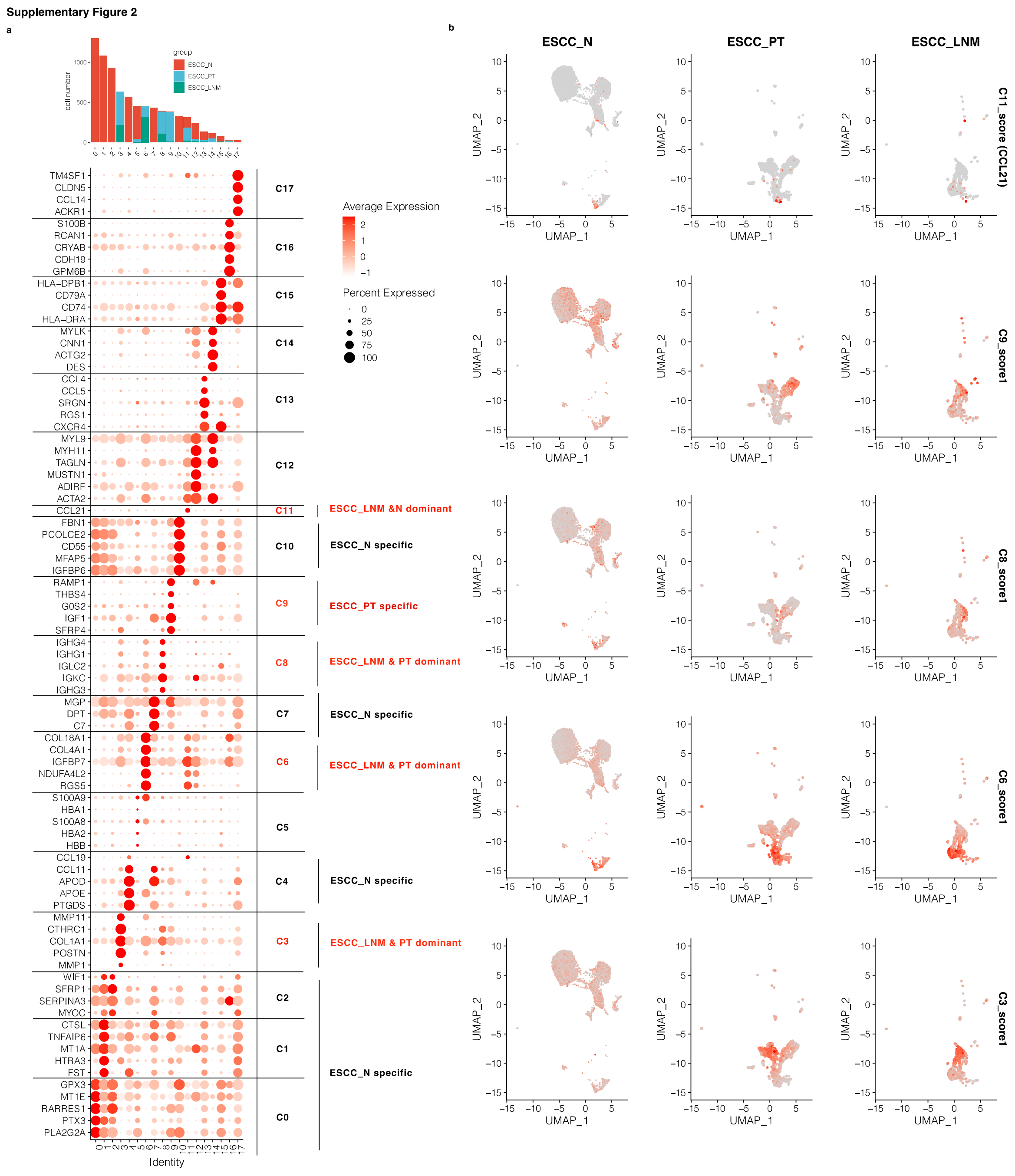

Figure S2. Fibroblast subclusters.

(a) Dot plot showing the differentially expressed genes (DEGs) among clusters of Fibroblast cells.

(b) The expression of the differentially expressed genes (DEGs) defined in (a) for fibroblast cells.

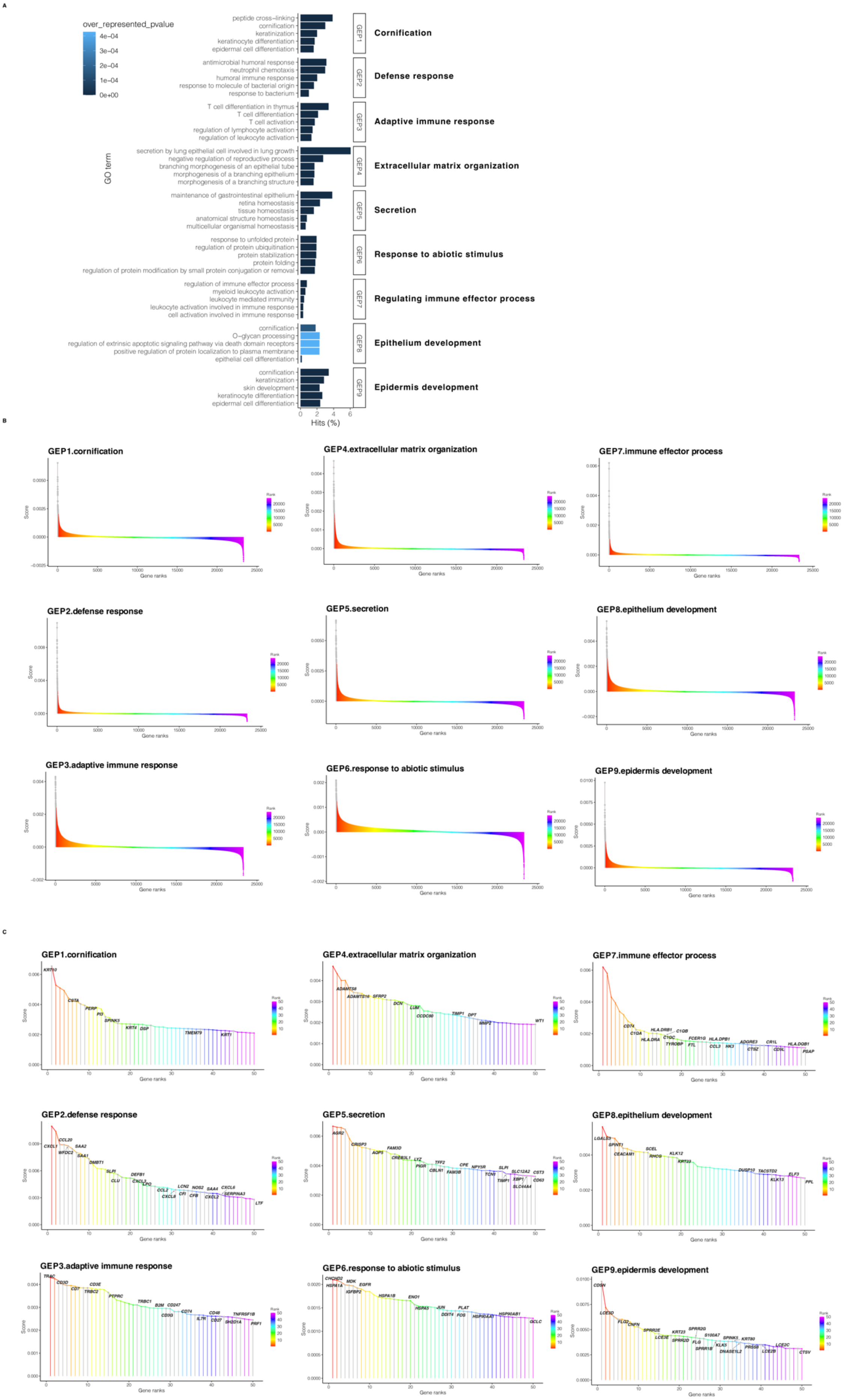
A
over_represented_pvalue
4e-04
3e-04
2e-04
1e-04
0e+00
GO term
peptide cross-linking
cornification
keratinization
keratinocyte differentiation
epidermal cell differentiation
antimicrobial humoral response
neutrophil chemotaxis
humoral immune response
response to molecule of bacterial origin
response to bacterium
T cell differentiation in thymus
T cell differentiation
T cell activation
regulation of lymphocyte activation
regulation of leukocyte activation
secretion by lung epithelial cell involved in lung growth
negative regulation of reproductive process
branching morphogenesis of an epithelial tube
morphogenesis of a branching epithelium
morphogenesis of a branching structure
maintenance of gastrointestinal epithelium
retina homeostasis
tissue homeostasis
anatomical structure homeostasis
multicellular organismal homeostasis
response to unfolded protein
regulation of protein ubiquitination
protein stabilization
protein folding
regulation of protein modification by small protein conjugation or removal
regulation of immune effector process
myeloid leukocyte activation
leukocyte mediated immunity
leukocyte activation involved in immune response
cell activation involved in immune response
cornification
O-glycan processing
regulation of extrinsic apoptotic signaling pathway via death domain receptors
positive regulation of protein localization to plasma membrane
epithelial cell differentiation
cornification
keratinization
skin development
keratinocyte differentiation
epidermal cell differentiation
Hits (%)
GEP1 Cornification
GEP2 Defense response
GEP3 Adaptive immune response
GEP4 Extracellular matrix organization
GEP5 Secretion
GEP6 Response to abiotic stimulus
GEP7 Regulating immune effector process
GEP8 Epithelium development
GEP9 Epidermis development
B
GEP1.cornification
GEP4.extracellular matrix organization
GEP7.immune effector process
GEP2.defense response
GEP5.secretion
GEP8.epithelium development
GEP3.adaptive immune response
GEP6.response to abiotic stimulus
GEP9.epidermis development
Score
Gene ranks
Rank
C
GEP1.cornification
GEP4.extracellular matrix organization
GEP7.immune effector process
GEP2.defense response
GEP5.secretion
GEP8.epithelium development
GEP3.adaptive immune response
GEP6.response to abiotic stimulus
GEP9.epidermis development
Score
Gene ranks
Rank

Figure S3. Annotation and characterization of 9 gene expression programs in Epithelial cells.

(a) Gene oncology analysis of top50 genes ranked by contribution score in each gene expression programs.

(b) The distribution of contributing score for all genes in each gene expression programs.

(c) The distribution of contributing score for top50 genes in each gene expression programs.

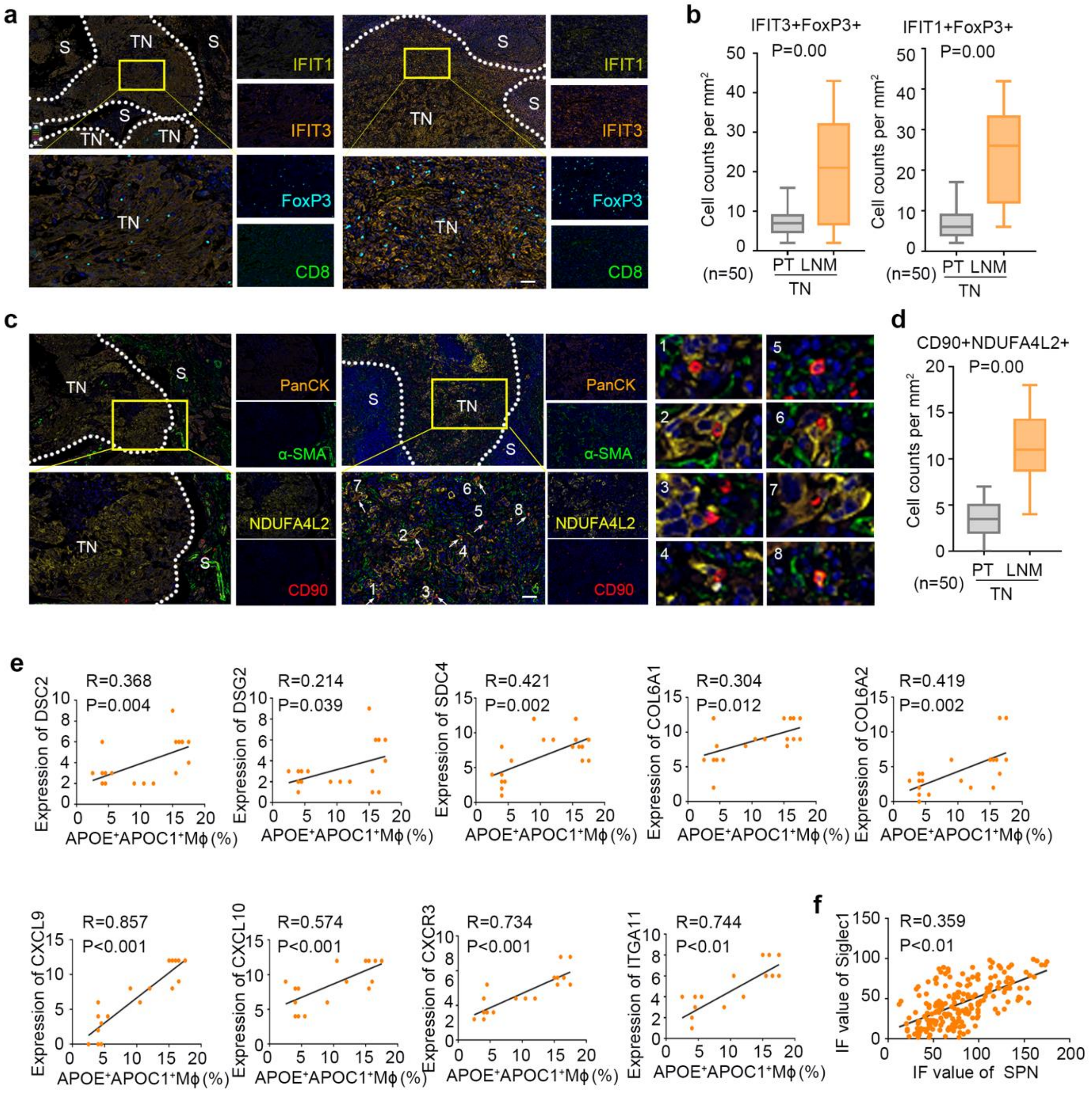

Supplementary Figure 4

Figure S4

(a) Representative images of multiplexed IF staining of IFIT1, IFIT3 and Foxp3 in primary tumor and metastatic lymph node (n=50). Scale bar, 50 μm.

(b) Percentage of IFIT1+FoxP3+, IFIT3+FoxP3+ T cells in tumor nest between primary tumor and metastatic lymph node.

(c) Representative images of multiplexed IF staining of Pan-CK, α-SMA, CD90 and NDUFA4L2 in primary tumor and metastatic lymph node (n=50). Scale bar, 50 μm. The white arrows indicate the location of CD90 and NDUFA4L2 positive cells.

(d) Density of CD90+NDUFA4L2+ CAFs in tumor nest between primary tumor and metastatic lymph node.

(e) Correlation between the expression of DSG2, DSG2, SDC4, COL6A1, COL6A2, CXCL9, CXCL10, CXCR3, ITGA11 and APOE+APOC1+ macrophages in metastatic lymph-node (n=20).

(f) The intensity of IF staining for Siglec1 and SPN in tumor (cell number n = 200) was analyzed with ImageJ software, and a linear regression analysis was performed to test their correlations. Data are presented as means ± SD. PT: primary tumor, LNM: metastatic lymph node.

Table S1. Clinical characteristic of patients recruited in this study.

Name	Gender	Age	Pathology	Grade	Vessel Invasion	Nerve Invasion	TNM	Stage
**P1**	Male	53	ESCC	G2	Yes	Yes	T3N2M0	III B
**P2**	Male	53	ESCC	G1	Yes	No	T3N1M0	III B
**P3**	Female	66	ESCC	G1	Yes	No	T3N1M0	III B
**P4**	Female	57	ESCC	G2	No	No	T1bN0M0	I B

Table S2. List of antibodies used in this project.

Antibody	Cat No.	Vendor	Application
**For IHC**			
**Rabbit anti-human APOE**	**ab279714**	**Abcam**	**IHC, 1:100**
**Rabbit anti-human**	ab1898	Abca	IHC: 1:100

**APOC1**	66	m	
**Rabbit anti-human COL6A1**	ab1514 22	Abca m	IHC, 1:200
**Rabbit anti-human COL6A2**	PA5-65085	Invitr ogen	IHC, 1:200
Mouse anti-human DSC2	ab218377	Abcam	IHC, 1:200
**Rabbit anti-human DSG2**	**ab9676 1**	**Abca m**	**IHC, 1:200**
Rabbit anti-human CXCL9	ab118543	Abcam	IHC, 1:200
Rabbit anti-human CXCL10	MA5-23774	Invitrogen	IHC, 1:200
Rabbit anti-human CXCR3	ab288437	Abcam	IHC, 1:200
Rabbit anti-human SDC4	GTX02062	GeneTex	IHC, 1:200
**For IF**			
Rabbit anti-human SIGLEC1	GTX131703	GeneTex	IF: 1:100
Mouse anti-human SPN	ab257315	Abcam	IF: 1:100
**For Multiplexed staining**			
Rabbit anti-human CD163	ab182422	Abcam	IF: 1:500
Rabbit anti-human CD68	ab213363	Abcam	IF: 1:1000
Rabbit anti-human PD-1	D4W2J, 86163S	CST	IF: 1:200
Rabbit anti-human PD-L1	E1L3N, 13684S	CST	IF: 1:400
Rabbit anti-human CD3	A0452	Dako	IF: 1:1
Rabbit anti-human CD4	ab133616	Abcam	IF: 1:100
Rabbit anti-human CD8	ab178089,	Abcam	IF: 1:200
Rabbit anti-human CD56	ab75813	Abcam,	IF: 1:1000
Rabbit anti-human CD20	L26, IR604	Dako	IF: 1:1
Rabbit anti-human FOXP3	ab20034	Abcam	IF: 1:100
**Rabbit anti-human APOE**	**ab1835 97**	**Abca m**	**IF: 1:100**
Rabbit anti-human APOC1	ab198288	Abcam	IF: 1:100
**Rabbit anti-human IFIT1**	**23247-1-AP**	**protein tech**	**IF: 1:100**
Rabbit anti-human IFIT3	15201-1-AP	proteintech	IF: 1:100
**Rabbit anti-human α-SMA**	**67 735-1-**	**protein tech**	**IF: 1:100**

	Ig		
Rabbit anti-human CD90	ab133350	Abcam	IF: 1:100
**Rabbit anti-human NDUFA4L2**	**16480-1-AP**	**proteintech**	**IF: 1:100**
Rabbit anti-human RGS5	11590-1-AP	proteintech	IF: 1:100
Rabbit anti-human pan-CK	ab7753	Abcam	IF: 1:100

Data S1. Metadata information of 85, 263 cells.

Data S2. Cluster signature genes (Top Rank 50).

Data S3. The expression level of cluster signature genes in the 95 patients from TCGA-ESCC cohort, inferred by the GSVA method based on bulk RNA-seq data.

Data S4. Top50 signature genes and their contribution score of 9 gene expression program in epithelial cells.

Data S5. The expression level of 9 epithelial gene expression program in the 95 patients from TCGA-ESCC cohort, inferred by the GSVA method based on bulk RNA-seq data.
